# Treatment strategy of unstable atlas fracture: A retrospective study of 21 patients

**DOI:** 10.1097/MD.0000000000020153

**Published:** 2020-05-01

**Authors:** Wei Guo, Yang Lin, Jingwen Huang, Feng Hu, Zhou Ding, Zengming Xiao

**Affiliations:** a Spine and Osteopathy Ward, The First Affiliated Hospital of Guangxi Medical University, Nanning, Guangxi; b Department of Spine Surgery, The First People's Hospital of Changde City, Hunan Province, P.R. China.

**Keywords:** fixing lateral mass, posterior transpedicular fixation, towel clamp, unstable atlas fracture

## Abstract

At present, the posterior cervical approach with open reduction and internal fixation (ORIF) remains a commonly effective treatment for unstable Atlas fracture. However, the inserted screws into the C1 lateral mass of some unstable atlas fracture are very difficult, so that the operation is forced to change into C0 to C2 fusion. In order to improve the successful rate of lateral mass screw placement, we introduced a method of fixing lateral mass with a towel clamp in posterior transpedicular fixation, and explore the efficacy and feasibility.

Twenty-one consecutive patients with unstable atlas fracture were treated via this method from October 2012 to July 2017. All cases had neck pain and restricted motion of neck movement on admission. Electronic medical records and pre- and postoperative radiographs were reviewed. Screw and rod placement, bone fusion, and spinal cord integrity were assessed via long-term follow-up with anteroposterior and lateral radiographs and computed tomography. Follow-up included clinical assessment of neurological function, assessment of pain using the visual analog scale (VAS), and assessment of the activities of daily living using the neck disability index (NDI).

The mean follow-up duration was 22.1 months (range: 12–54 months). No screw loosening or breakage, plate displacement, neurovascular injury, and severe complications occurred during follow-up. The mean operative time was 112.4 ± 14.9 min (range: 82–135 min), and mean blood loss was 386.2 ± 147.9 mL (range: 210–850 mL). One patient experienced continuous neck pain postoperatively, but this gradually disappeared with analgesic administration. At final follow-up, all patients had bone fusion, the VAS scores and NDI were significantly improved compared with preoperatively.

Fixing the C1 lateral mass with a towel clamp during posterior transpedicular fixation for unstable atlas fracture appears to be a safe and reliable method, with the advantages of being a simple technique with few complications.

## 1 Introduction

Atlas fracture is rare, accounting for 3% to 13% of all cervical spine fractures, and 1.3% to 2% of all spinal injuries.^[1,2]^ Unstable atlas fracture is even rarer, and is mainly caused by vertical falls, traffic accidents, and other vertical traumatic force. Unstable atlas fracture comprises an atlas burst fracture with simultaneous injury to the transverse atlantal ligament (TAL), characterized by outward displacement of the lateral masses, and atlantoaxial dislocation or subluxation. Unstable atlas fracture is very dangerous, as the displacement of bone blocks may cause spinal cord injury, leading to severe complications such as paraplegia and death. Hence, the stability of the atlantoaxial complex must be surgically reconstructed. Treatment aims to correct the dislocation, restore the stability of the atlantoaxial joint, and retain the maximum degree of motion of the cervical spine.

The treatment of unstable atlas fracture remains controversial, and there is a lack of uniform standards or guidelines.^[3]^ Studies have described types of surgery performed for unstable atlas fractures, such as transoral approach anterior C1-ring plate osteosynthesis, posterior osteosynthesis with a lateral mass screw rod, and posterior C1 to C2 fusion and C0 to C2 fusion. However, anterior C1-ring plate osteosynthesis is prone to postoperative infection, and posterior osteosynthesis with a lateral mass screw rod can cause incomplete reduction of C1 anterior arch fractures; hence, these surgical methods are not widely used.^[1,4]^ Although posterior fusion eliminates the mobility of the cervical spine, it is still useful for some types of unstable atlas fracture.

Posterior C1 to C2 fusion with transpedicular fixation has the advantages of reliable fixation, few complications, and high fusion rates, while retaining part of the rotation and flexion extension; therefore, it is the most commonly used method for unstable atlas fracture.^[5,6]^ However, it is very difficult to fix the atlantoaxial joint in some unstable atlas fractures, as the unstable lateral mass swings or moves forward during the insertion of screws into the C1 lateral mass. Therefore, it may be necessary to perform C0 to C2 fusion instead of C1 to C2 fusion, which may cause other complications such as loss of activity of the atlantooccipital joint. To increase the success rate of screw insertion into the C1 lateral mass in unstable atlas fractures, we introduced a method in which a towel clamp is used to fix the lateral mass while the pedicle screw is inserted into the lateral mass. We consider that this method makes surgery easier and safer for displaced unstable atlas fractures. The present study aimed at to evaluate the safety and effectiveness of this novel method.

## 2 Materials and methods

### 2.1 Clinical data

This retrospective study was approved by the ethical review committee of our institution. Twenty-one consecutive patients (17 males, 4 females) were diagnosed with unstable atlas fractures and treated surgically in our institution between October 2012 to December 2017 (Flow diagram). Inclusion criteria were

1. inpatient due to trauma,
2. suffered from C1 fracture (type III and IV),
3. treated with the fixed lateral mass technique before inserting the screw,
4. availability of radiographic examinations (cervical X-ray, CT, and MRI) and clinical data (inpatient electronic medical records and questionnaire).

Exclusion criteria were

1. suffered from C1 fracture (type I, II, and V fracture),
2. no surgery or incomplete date,
3. associated with occipital condyle and odontoid fracture,
4. other comorbidities, such as cervical stenosis, neoplasia, and infection.

All cases were treated with posterior transpedicular fixation; a towel clamp was used to fix the lateral mass, and the pedicle screw was then inserted into the lateral mass. Unstable atlas fractures associated with occipital condyle and odontoid fractures were excluded. The electronic medical records and pre- and postoperative radiographs of each patient were reviewed. Mean patient age was 56.5 years (range 22–71 years). The causes of atlas fracture were: injury during a fall (n = 12), motor vehicle accident (n = 8), and injury by falling objects (n = 1). All patients had neck pain and stiffness with or without pain in other parts. Except for one patient in a transient coma, the rest were awake, cooperative, and had no neurological deficits at presentation. One patient also had C4/C5 traumatic disc herniation, while another had T4 burst fracture (Table 1).

**Table 1:**
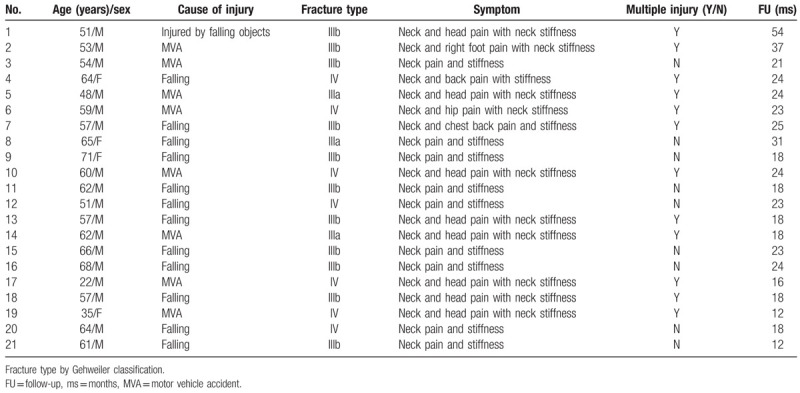
Patient demographic and baseline characteristics.

### 2.2 Preoperative care

Skull traction was performed in each case for 2 weeks, with a weight of 2 kg and an appropriate angle in accordance with the individual mechanism of injury. All patients received routine perioperative antibiotics 30 min before surgery. Patients were placed in prone position in a Mayfield head holder. The head was placed in the neutral position. The skin of the posterior part of the neck was not wrinkled by pulling the shoulders with elastic adhesive bandages; this was helpful in obtaining good exposure of the posterior arch of the atlas, and contributed to partial reduction.

### 2.3 Surgical technique

**Figure 1:**
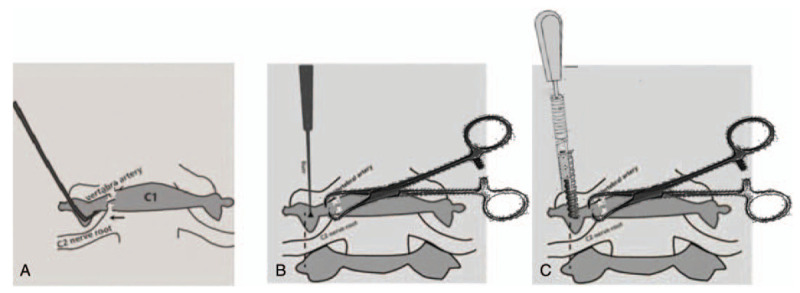
Illustration of the notching procedure and screw placement after the lateral mass was fixed. (A) A hook was used to palpate along the vertebral surface of the posterior arch from inside to outside to determine the medial border of the C1 pedicle and the entry point of the vertebral pedicle screw. (B) A large towel clamp was used to fix the lateral mass, and the notching procedure was then performed. (C) The screw was inserted into the lateral mass of the atlas.

**Figure 2:**
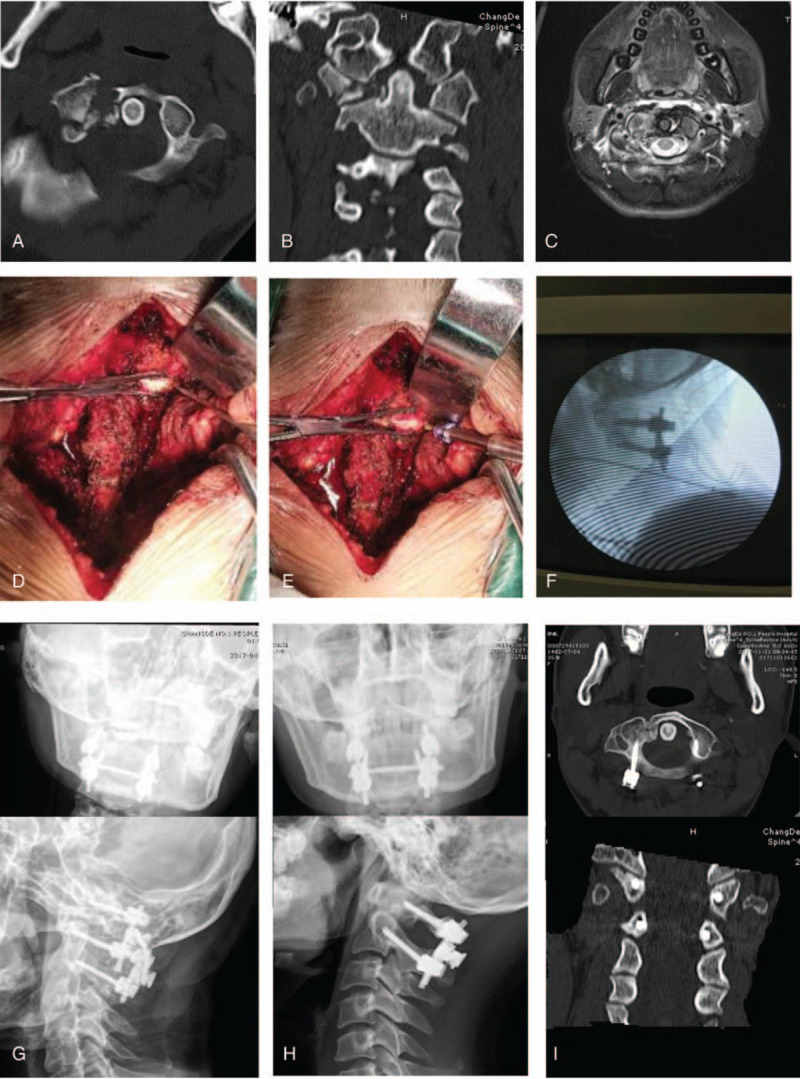
Pre-, intra-, and postoperative imaging of a 35-year-old female patient with Gehweiler classification type III atlas fracture. (A) and (B) Axial and coronal three-dimensional CT reconstruction images showed anterior and posterior arch fractures, and lateral mass burst fracture and displacement. (C) Axial magnetic resonance images showed partial transverse atlantal ligament rupture. (D) and (E) Intraoperative photographs showing the use of a large towel clamp to fix the lateral mass, then notching and screw insertion into the lateral mass of the atlas. (F) C-arm fluoroscopy was used to determine the position of the internal fixation. (G) and (H) Anteroposterior and lateral radiography showing fracture reduction and internal fixation positioning immediately postoperatively, and 3 months postoperatively. (I) Three-dimensional CT reconstruction at 6 months postoperatively showed fracture union.

A standard posterior midline incision was made from the occipital tubercle to the C3 spinous process. The posterior arches of the C1 and C2 laminae were then exposed subperiosteally to the external edge of the articular process. A hook was used to palpate along the vertebral surface of the posterior arch from inside to outside to determine the medial border of the C1 pedicle and the entry point of the vertebral pedicle screw. After confirming the entry point, a large towel clamp was used to fix the lateral mass, and the screw was then inserted into the C1 lateral mass using the notching technique.^[7,8]^ Fixing the lateral mass prevented the lateral mass from swinging or moving forward (Fig. 1). Next, a pedicle screw was placed into the C2 pedicle, and a titanium rod was used to connect the lateral mass screw with the C2 pedicle screw; this helped in the process of notching and placing screws on the contralateral side. In the same way, pedicle screws were placed in the contralateral lateral mass and C2 pedicle. The next step was to tighten the nut, and connect and fix the titanium rod on the contralateral side; the nut on the first side was then loosened and refastened, so that the fracture could be reset and the dislocation could be corrected. The mean lengths of the C1 lateral mass screw and C2 pedicle screw were 26 and 28 mm, respectively. Satisfactory fracture reduction and correction of atlantoaxial dislocation was confirmed on intraoperative C-arm fluoroscopic view or postoperative CT three-dimensional imaging (Fig. 2).

### 2.4 Postoperative treatment

Patients were mobilized on postoperative day 1. The drainage tube was removed when the drainage fluid was <30 mL/day. Routine prophylactic antibiotics were administered for up to 48 h postoperatively. External immobilization via a hard collar was used for 6 weeks postoperatively.

### 2.5 Follow-up

CT or anteroposterior and lateral radiography was performed within 1 to 2 weeks postoperatively to assess the accuracy of screw placement and the effectiveness of fracture reduction. All patients were followed up at postoperative 6 weeks, 3 months, and 12 months to evaluate pain via the VAS, NDI, and neurological function. Three-dimensional CT was performed at 6 months postoperatively to evaluate bone fusion and fracture healing.

### 2.6 Statistical methods

All data were analyzed by SPSS (Statistical Package for the Social Sciences) version 19. Data were expressed as mean ± standard deviations for variables. Preoperative, post-operative 1 week and the final follow-up differences were performed using a paired *t* test, and statistical significance was set at *P* < .05.

## 3 Results

In all cases, postoperative radiography showed that satisfactory surgical reduction was achieved. A total of 21 plates were placed, and all 42 screws were accurately inserted into the atlas lateral masses. Mean operative time was 112.4 ± 14.9 min (range: 82–135 min), and mean blood loss was 386.2 ± 147.9 mL (range: 210–850 mL) (Table 2). No patient required blood transfusion. There were no complications such as neurological deficits, vertebral artery injury, and/or wound infection. Mean follow-up duration was 22.1 months (range: 12–54 months). Mean VAS score was 6.6 ± 1.3 (range: 4–8) preoperatively, 2.1 ± 0.7 (range: 1–3) postoperatively, and 1.0 ± 0.7 (range: 0–2) at final follow-up. Mean NDI was 39.4 ± 3.8 (range: 33–45) preoperatively, 23.6 ± 2.3 (range: 20–27) postoperatively, and 5.3 ± 1.2 (range: 3–8) at final follow-up. The VAS and NDI scores at postoperative and final follow-up were significantly improved compared with those before operation (*P* < .05) (Table 3). One patient had continuous neck pain postoperatively, but this gradually disappeared with analgesic administration. During follow-up, there was no screw loosening or breakage, plate displacement, and/or other complications. CT confirmed that bony healing and bone fusion of the fractures were achieved at 6 or 12 months postoperatively (Table 2).

**Table 2:**
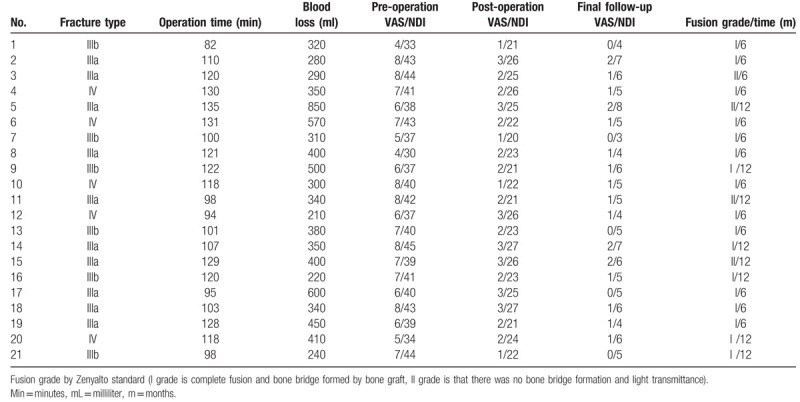
Clinical and imaging characteristics in pre-/post-operation.

**Table 3:**
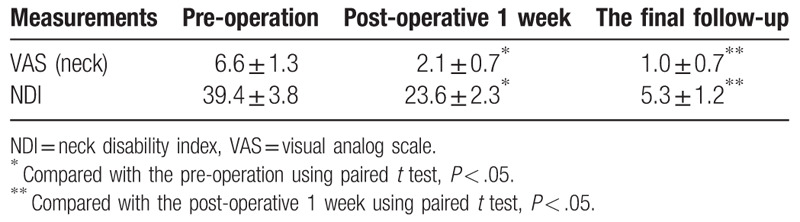
VAS and NDI of pre-operation, post-operative 1 week and the final follow-up in 21 patients.

## 4 Discussion

The atlas is different from other vertebral bodies, as it is a ring structure that lacks a vertebral body and spinous process; it consists of an anterior arch, posterior arch, and two lateral masses. The middle canal formed by the ring structure of the atlas is part of the spinal canal, with the spinal cord, dura mater, and dural membrane passing through it. The anterior tubercle is located anterior to the anterior arch, and is the attachment point of the anterior longitudinal ligament and the longus colli muscles. The surfaces of the longus colli muscles are covered with the tectorial membrane, which is composed of dense connective tissue. Posterior to the anterior tubercle is the fovea dentis, which is the articulation point for the odontoid process of C2. The rounded edge of the posterior arch is attached to the posterior atlantooccipital membrane, which is a cranial extension of the highly elastic ligamentum flavum. The groove formed at the junction of the posterior arch and the lateral mass is the sulcus arteriae vertebralis, through which the vertebral artery and the first spinal nerve pass.^[9–11]^

The anatomy of the region adjacent to the atlas is complex. The articular surface above the lateral mass is connected with the occipital condyle by the atlantooccipital joint, and the lower articular surface is connected with the axis by the atlantoaxial joint.^[9]^ The odontoid process and its anterior fovea dentis constitute the atlantoodontoid joint, which forms an intracapsular joint with the posterior TAL. The TAL attaches to the medial tubercle of the C1 lateral mass. The atlantoaxial joint, atlantoodontoid joint, intraarticular ligament, and surrounding muscles and ligaments form a complicated atlantoaxial joint complex. The stability of the atlantoaxial complex is maintained by the joint capsule ligament, anterior longitudinal ligament, tectorial membrane, alar ligament, apical odontoid ligament, TAL, longitudinal bands of the cruciate ligament, and surrounding muscles and ligaments. The posterior interspinous ligament, supraspinal ligament, and occipital neck muscle enhance the stability of the atlantoaxial complex.

There are several classification systems for atlas fractures, but none is uniformly accepted. The Jefferson, Landells, and Gehweiler classifications are most commonly used in clinical studies.^[12,13]^ Jefferson classified atlas fractures into five types, including anterior arch, posterior arch, burst, lateral mass, and lateral mass plus posterior arch fractures.^[2]^ Landells and Van Peteghem proposed another classification system in which posterior and anterior arch fractures were defined as type I, bilateral posterior arch fracture with unilateral or bilateral anterior arch fracture as type II, and a lateral mass fracture with or without a posterior arch fracture as type III.^[12]^ On the basis of the Jefferson classification system, Gehweiler divided atlas fractures into five types: type I (isolated fracture of the anterior arch); type II (isolated bilateral fracture of the posterior atlas ring); type III (fracture of both arches of the atlas, corresponding to the “Jefferson fracture”), which was further divided into type IIIa (intact TAL), and type IIIb (ruptured TAL)^[14]^; type IV (involving the lateral mass); and type V (isolated fractures of the C1 transverse process).^[13]^

The TAL plays a crucial role in atlantoaxial stability, with atlas fractures often defined as stable or unstable based on the inferred integrity of the TAL. Dickman defined an intraligamentous rupture as Dickman type I, and bony avulsion as Dickman type II.^[15]^ The “rule of Spence” suggests that the TAL is probably torn in burst atlas fracture with displacement of the lateral masses of more than 6.9 mm; however, displacement of the lateral mass of <6.9 mm or without displacement of the TAL is likely to be overlooked, as plain radiography and CT do not provide direct visualization of the TAL.^[16,17]^ The integrity of the TAL can be evaluated with high resolution magnetic resonance imaging. This is important in distinguishing between stable and unstable burst fractures.^[18]^

At present, the treatment of C1 fractures remains controversial, and there are no internationally accepted treatment standards. For isolated atlas fractures, conservative treatment is the main method, but for unstable atlas fractures, surgical treatment is still the preferred method.^[19]^ It is widely accepted that surgery is indicated for atlas fracture associated with atlantooccipital instability, intraligamentous rupture of the TAL, and for “unstable” atlas fracture.^[3,20]^ At present, there are several surgical methods with osteosynthesis for the treatment of unstable fractures, such as transoral approach anterior C1-ring plate osteosynthesis, posterior osteosynthesis with a lateral mass screw rod, and posterior C1 to C2 fusion and C0 to C2 fusion.^[21–23]^ However, any surgical method has its advantages and disadvantages. For example, transoral approach anterior C1-ring plate osteosynthesis can retain important joint motion, but it can only repair of anterior 1/2 Jefferson fractures and has a high risk of wound infection; posterior osteosynthesis with a lateral mass screw rod can preserve rotary motion and prone to lower postoperative infection, but it is unable to stabilize fractured anterior arch and cause incomplete reduction of C1 anterior arch fractures; C0 to C2 fusion seems relatively stable, but its internal fixation shear force is larger, and it is prone to complications such as screw loosening and internal fixation failure. However fortunately, C1 to C2 fusion absorbs the advantages of the three operations mentioned above. It has reliable reduction, preserves the activity of the atlanto-occipital joint and is suitable for various unstable Jefferson fractures with lower risk of would infection, is a satisfactory treatment for unstable Jefferson fractures. Recent reports indicate that definitive atlantoaxial fusion is the current treatment of choice for elderly patients with unstable Gehweiler type 3b atlas fracture, as elderly patients have reduced bony healing ability, and isolated atlas osteosynthesis is not recommended in type 3b fractures with severe dislocated bony avulsion of the TAL.^[2,24]^ This report provides a reference for the treatment of unstable Jefferson fracture.

In the posterior approach for unstable atlas fracture, it is necessary to place a screw in the C1 lateral mass, irrespective of whether the fracture is being treated via osteosynthesis or atlantoaxial fusion. For Gehweiler type III atlas fractures and obviously displaced lateral mass fractures, the lateral mass swings or moves forward during C1 screw placement; this increases the risk of injury to the spinal cord and vertebral artery, which are potentially fatal complications. We use a towel clamp to fix the lateral mass, then drill the hole and insert the screw. The fixed position of the towel clamp is located at the stump of the posterior arch, with one side adjacent to the fracture line, the other side close to the lateral mass screw placement, and the upper edge adjacent to the vertebral artery. This technique is very important to temporarily stabilize the lateral mass during screw placement. It improves the success rate of screw placement in the C1 lateral mass, and avoids the implementation of C0 to C2 fusion and complications such as loss of activity of the atlantooccipital joint after fusion. It also reduces the risk of damaging important organs such as the spinal cord and the vertebral artery. In addition, it makes the operation easier, resulting in less damage and less bleeding.

In the present study, 21 patients with an unstable atlas fracture were treated with C1 to C2 pedicle screw fixation and fusion after C1 lateral mass fixation with a towel clamp. The postoperative VAS and NDI was significantly lower than preoperatively. At final follow-up, three-dimensional CT showed satisfactory C1 to C2 bone fusion, with no broken screws, broken rods, and/or lateral mass separation. However, one patient had postoperative neck pain that was resolved after analgesia and neurotrophic treatment. There were two possible reasons for this pain. One is that in cases involving excessive traumatic force, atlantoaxial instability leads to cervical muscle spasm and severe edema after muscle and ligament damage, which stimulates the occipital nerve to cause pain; another is that there was no subperiosteal dissection during the surgical incision of the muscle, which may damage the occipital nerve or branch.

The primary limitation of the present study is the small sample size. As this method is used in more cases, its safety and efficacy may be more thoroughly evaluated. Another limitation is the retrospective design; future prospective studies may better control for follow-up timing intervals, and could include more standardized outcome measures.

## 5 Conclusion

In conclusion, posterior transpedicular fixation while the lateral mass is fixed with a towel clamp is safe and reliable during treatment of unstable atlas fracture. This method is simple, enables effective fracture reduction, high fusion rates, and has few complications.

## Author contributions

**Conceptualization:** Jingwen Huang, Zengming Xiao.

**Data curation:** Zhou Ding.

**Formal analysis:** Wei Guo, Feng Hu.

**Funding acquisition:** Zengming Xiao.

**Investigation:** Wei Guo, Zhou Ding.

**Methodology:** Wei Guo, Yang Lin.

**Resources:** Wei Guo, Zengming Xiao.

**Software:** Wei Guo.

**Supervision:** Jingwen Huang, Feng Hu, Zengming Xiao.

**Validation:** Wei Guo, Zengming Xiao.

**Visualization:** Zengming Xiao.

**Writing – original draft:** Wei Guo, Yang Lin.

**Writing – review & editing:** Zengming Xiao.
